# FGF21 is required for protein restriction to extend lifespan and improve metabolic health in male mice

**DOI:** 10.1038/s41467-022-29499-8

**Published:** 2022-04-07

**Authors:** Cristal M. Hill, Diana C. Albarado, Lucia G. Coco, Redin A. Spann, Md Shahjalal Khan, Emily Qualls-Creekmore, David H. Burk, Susan J. Burke, J. Jason Collier, Sangho Yu, David H. McDougal, Hans-Rudolf Berthoud, Heike Münzberg, Andrzej Bartke, Christopher D. Morrison

**Affiliations:** 1 Pennington Biomedical Research Center, Baton Rouge, LA 70808 USA; 2 Department of Internal Medicine-Geriatrics Laboratory, Southern Illinois University School of Medicine, Springfield, IL 62702 USA

**Keywords:** Ageing, Metabolic syndrome

## Abstract

Dietary protein restriction is increasingly recognized as a unique approach to improve metabolic health, and there is increasing interest in the mechanisms underlying this beneficial effect. Recent work indicates that the hormone FGF21 mediates the metabolic effects of protein restriction in young mice. Here we demonstrate that protein restriction increases lifespan, reduces frailty, lowers body weight and adiposity, improves physical performance, improves glucose tolerance, and alters various metabolic markers within the serum, liver, and adipose tissue of wildtype male mice. Conversely, mice lacking FGF21 fail to exhibit metabolic responses to protein restriction in early life, and in later life exhibit early onset of age-related weight loss, reduced physical performance, increased frailty, and reduced lifespan. These data demonstrate that protein restriction in aging male mice exerts marked beneficial effects on lifespan and metabolic health and that a single metabolic hormone, FGF21, is essential for the anti-aging effect of this dietary intervention.

## Abstract (web summary)

The restriction of dietary protein or amino acid intake is well established to extend lifespan in multiple species. Here, the authors show that the endocrine hormone FGF21 is necessary for dietary protein restriction to extend lifespan and improve metabolic health in aged, male mice.

## Introduction

A variety of dietary interventions (i.e., calorie restriction, intermittent fasting, fasting mimetics, and dietary restriction) improve health and lifespan^1,2^. Epidemiological data suggest that lowering dietary protein content supports metabolic improvements and resilience^3–7^, while high protein intake correlates with increased mortality^8,9^. Protein restriction (PR) is a form of dietary restriction in the absence of energy restriction that extends lifespan and improves general health measures in various organisms, including rodents, fruit flies, and yeast^10–15^. The restriction of protein but not the restriction of fat or carbohydrate increases lifespan in fruit flies^16,17^. In rodents, PR also extends lifespan^18–21^, with evidence suggesting that lowering protein consumption exerts favorable outcomes on health that are independent of energy intake^18^. As an alternative to total PR, the restriction of select amino acids^22^, including methionine restriction^23,24^, threonine and/or tryptophan restriction^25–27^, and branched-chain amino acid (BCAA) restriction^28–31^, also extend lifespan in various organisms. Collectively, these data suggest that protein/amino acid restriction may engage unique mechanisms that improve metabolic health and extend lifespan.

The PR-induced improvements on health naturally create an interest in the underlying cellular mechanisms. Most work has accentuated the ability of protein or amino acid restriction to engage a host intracellular nutrient-sensing pathways, including mTOR, GCN2, AMPK, autophagy, etc^12,32–39^. However, several years ago our lab hypothesized that an endocrine effector signal of protein restriction might exist. This focus led to the discovery that the liver-derived hormone FGF21 is robustly induced by PR and that the deletion of FGF21 blocks adaptive metabolic responses to PR in young mice^40–45^. FGF21 is a pleiotropic hormone, with circulating levels primarily being produced by the liver^46^. FGF21 signals through fibroblast growth factor receptors (FGFRs), but binds with low affinity such that proper signal transduction requires interaction with the co-receptor beta-Klotho (Klb)^47^. FGF21 increases energy expenditure, enhances glucose metabolism, and upregulates the thermoregulatory marker UCP1^48^. FGF21 also crosses the blood-brain barrier^49^, and several studies suggest that the physiological effects of FGF21 are mediated by the brain^50,51^. Recent data from our lab indicate that FGF21/Klb signaling in the brain is essential for PR to increase energy expenditure, improve glucose homeostasis, and protect against diet-induced obesity in young mice^45^.

Factoring in FGF21’s key role in facilitating the metabolic response to PR in young mice, and that transgenic overexpression of FGF21 extends lifespan and improves insulin sensitivity^52^, we hypothesized that increases in FGF21 might mediate the beneficial effects of long-term PR in aging animals. Here we demonstrate that, in male mice, FGF21 is required for the effects of PR on lifespan and metabolism. Indeed, mice that are FGF21 deficient are not only resistant to the health benefits effects of PR, but they also exhibit early-onset weight loss, increased frailty, and reduced lifespan when fed a low protein diet. Collectively, these data represent a suggest that FGF21 is essential for the pro-longevity effects of PR and highlight the power of a single endocrine hormone to coordinate metabolic and behavioral responses that improve metabolism and longevity.

## Results

### FGF21 is essential for increasing lifespan during dietary protein restriction

**Fig. 1:**
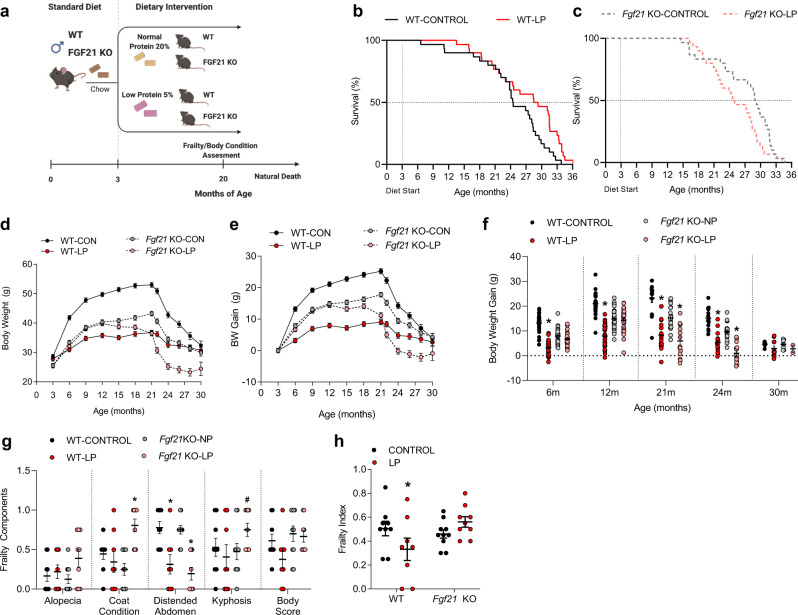
Deletion of FGF21 reverses the beneficial effects of dietary protein restriction on lifespan. **a** Schematic representation of the longevity study. Wildtype and *Fgf21*-KO mice were placed on control or LP diet at 3 months of age and monitored until natural death. **b** Kaplan–Meier survival curve for wildtype mice on LP diet, indicating an extension of lifespan in WT mice on LP. *n* = 30 per group. **c** Kaplan–Meier survival curve for *Fgf21*-KO mice on LP, indicating a reduction in lifespan in *Fgf21* KO mice on LP. *n* = 30 mice per group. **d**, **e** Bodyweight and bodyweight gain curves based on the initiation of diets, group*time *p* = 0.001. **f** Comparison of body weight change at specific time points, group*time *p* = 0.001. **g** Specific components were used from the Howlett Frailty Index assessed at 20 months of age, *n* = 9–10 mice per group; alopecia geno*diet *p* = 0.2115, coat condition geno*diet *p* = 0.0009, distended abdomen diet *p* = 0.0001, kyphosis geno*diet *p* = 0.09, body score geno*diet *p* = 0.2854. **h**, Frailty Index as an average from individual components in g, geno*diet *p* = 0.02. Statistical analyses were conducted using survival analysis for lifespan and one-way or two-way ANOVA for BW change and Frailty. Values are mean ± SEM, with significant posthoc comparison within the diet*genotype interaction noted as *p < 0.05 and ^#^*p* < 0.10 compared with respective control. Source data are provided as a Source Data file.

To test FGF21’s role in mediating the longevity effects of long-term dietary PR, 60 male C57BL/6 J (control wild-type) mice and 60 male *Fgf21* KO mice were placed on either control (CON) or low protein (LP) diet, generating four groups of 30 mice each (Fig. 1a). Mice consumed these diets ad libitum from 3 months of age until natural death. We observed that the LP diet extended lifespan in wild type (WT) mice (Kaplan-Meier survival analysis; *p* = 0.01365; Fig. 1b). However, exposing *Fgf21* KO mice to the LP diet reduced lifespan (*p* = 0.044; Fig. 1c). Parametric Survival Fit using a 2-way analysis (Diet, Genotype, G*D) resulted in a significant diet*genotype interaction (*p* = 0.0107). Cox Proportional Hazards analysis also results in a significant diet*genotype interaction (*p* = 0.002), with LP reducing the risk ratio in WT mice (RR = 0.49 for WT-LP vs WT-CON, *p* = 0.008) but increasing the risk ratio in *Fgf21* KO mice (RR = 1.60 for *Fgf21* KO-LP vs *Fgf21* KO-CON; *p* = 0.072). Unexpectedly, a trend was observed for KO-CON mice to live longer than WT-CON mice, although the effect did not reach statistical significance (Survival analysis Log-Rank *P* = 0.0978; Cox Proportional Hazards Fit *P* = 0.0838).

The changes in lifespan in response to LP were reflected by distinctive changes in body weight gain over time. WT-CON mice exhibited the expected increase in BW through approximately 21 months of age, followed by a progressive age-related BW decline during the third year of life (Fig. 1d–f). WT-LP mice exhibited reduced body weight gain through the first two years of life, with a slightly delayed and less severe loss of body weight over the final year. *Fgf21* KO-CON mice weigh less than WT-CON mice, yet age-related changes in body weight were similar to WT-CON mice, with weight peaking at approximately 21 months and then declining throughout the final year. *Fgf21* KO-LP mice, in contrast, do not initially alter body weight in response to the LP diet. However, age-related weight loss occurred much earlier (~15–18 months vs 22 months), such that *Fgf21* KO-LP mice weigh less than even WT-LP mice at ~26 months.

In both mice and humans, the prevalence and extent of frailty increase with age^53^. Recent data suggest that dietary interventions such as CR and BCAA restriction reduce the incidence of frailty in mice^31^. To examine the effects of FGF21 during LP feeding on additional geriatric measures, we adapted an analysis of frailty from the Howlett Frailty Index, including the individual factors alopecia, coat condition, distended abdomen, kyphosis, and body score (Fig. 1g, h). At 22 months of age LP diet tended to reduce frailty markers in WT mice but increased them in *Fgf21* KO mice. As a result, an average across all scores (Fig. 1h) demonstrated a significant diet*genotype interaction (*p* = 0.02), with LP reducing frailty in the WT but not the *Fgf21* KO mice.

Collectively, these data suggest that the interaction of FGF21 and PR reduces body weight gain, reduces frailty, and extends lifespan in mice. Conversely, *Fgf21* KO mice do not exhibit initial changes in body weight in response to the LP diet, and then beyond ~12 months of age the LP diet begins to exert an adverse effect in *Fgf21* KO mice, including earlier onset of age-related weight loss, increases in some frailty markers, and shortened lifespan.

### FGF21 mediates low protein-induced changes in BW gain, food intake, and body composition during aging

**Fig. 2:**
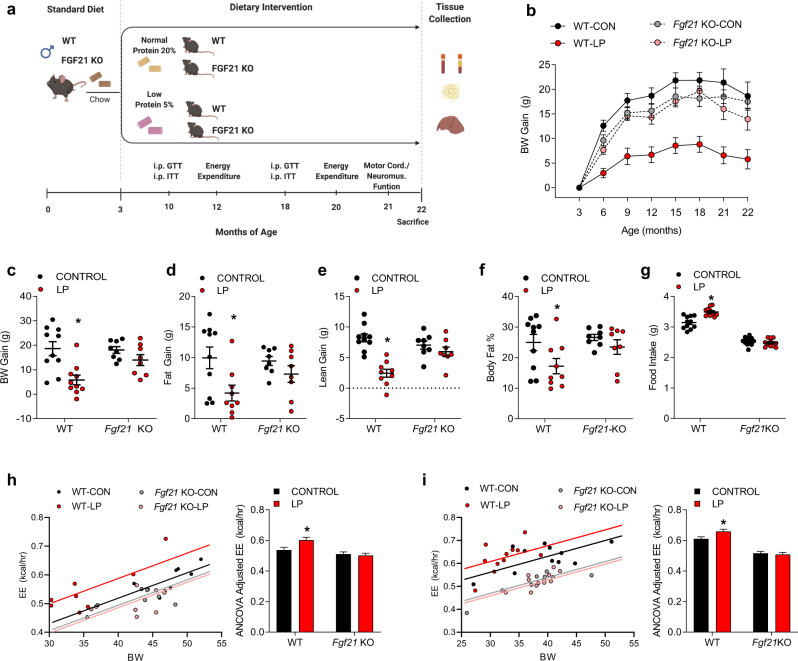
FGF21 mediates low protein-induced changes in BW gain, food intake, and body composition in aged mice. **a** Schematic representation of the metabolic aging study. Wildtype and *Fgf21*-KO mice were placed on control or LP diet at 3 months of age (12 mice/group), with various metabolic endpoints assess throughout the study as indicated, and tissue collection at 22 months of age. **b** Body weight gain over time following initiation of experimental diets, *n* = 12 mice/group, group*time *p* = 0.001. **c** Final body weight gain at 22 months of age (*n* = 8–12 mice/group at sacrifice; geno*diet *p* = 0.0004). **d** Final body fat gain at 22 months of age as measured by NMR (*n* = 8–12 mice/group at sacrifice; geno*diet *p* = 0.03). **e** Final body lean gain at 22 months of age as measured by NMR (*n* = 8–12 mice/group at sacrifice; geno*diet *p* = 0.0001). **f** Percent body fat at 22 months of age as measured by NMR (*n* = 8–12 mice/group at sacrifice; geno*diet *p* = 0.03). **g** Average daily food intake throughout the study (*n* = 8–12 mice/group at sacrifice; geno*diet *p* = 0.0001). **h** Energy expenditure (ANCOVA adjusted for BW) as measured at 12 months of age (*n* = 8–12 mice/group at sacrifice; geno*diet *p* = 0.04). **i** Energy expenditure (ANCOVA adjusted for BW) as measured at 20 months of age (*n* = 8–12 mice/group at sacrifice; geno*diet *p* = 0.04). Statistical analyses were conducted using two-way ANOVA or ANCOVA. All values are mean ± SEM, with significant posthoc comparison within the diet*genotype interaction noted as *p < 0.05 compared with respective control. Source data are provided as a Source Data file.

The above data demonstrate that lifespan extension due to PR requires FGF21. To test whether FGF21 contributes to the effects of long-term PR on healthspan in aged mice, a separate group of WT and *Fgf21 KO* mice were fed a control or LP diet from 3 months to 22 months of age (*n* = 12 mice per diet/genotype in 4 total groups). Metabolic and behavioral endpoints were then assessed throughout the experiment as outlined in Fig. 2a. As expected, the LP diet reduced BW gain in WT mice (Fig. 2b, c; *p* < 0.05), while this effect was absent in *Fgf21 KO* mice. Body composition analysis indicates that LP diet reduced both fat and lean gain in WT mice (Fig. 2d, e; *p* < 0.05), but that the reduction in fat gain was larger than that of lean gain, such that fat mass relative to body weight (percent body fat) was reduced in WT-LP mice compared to controls (Fig. 2f; *p* < 0.05). All of these LP-induced changes in body composition were absent in *Fgf21 KO* mice. Food intake was persistently elevated in WT-LP mice (*p* < 0.05), but again this effect was absent in *Fgf21 KO* mice (Fig. 2g). Lastly, energy expenditure (EE), adjusted for BW via ANCOVA, was increased by LP diet at both 12 and 20 months of age in WT mice (Fig. 2h, i, *p* < 0.05). Conversely, there was no increase in EE in *Fgf21 KO* mice on LP. These data suggest that dietary protein restriction persistently reduces body weight but increases food intake and energy expenditure in aging mice, with these effects depending on increased circulating FGF21.

### Protein restriction protects against age-related functional decline via FGF21

**Fig. 3:**
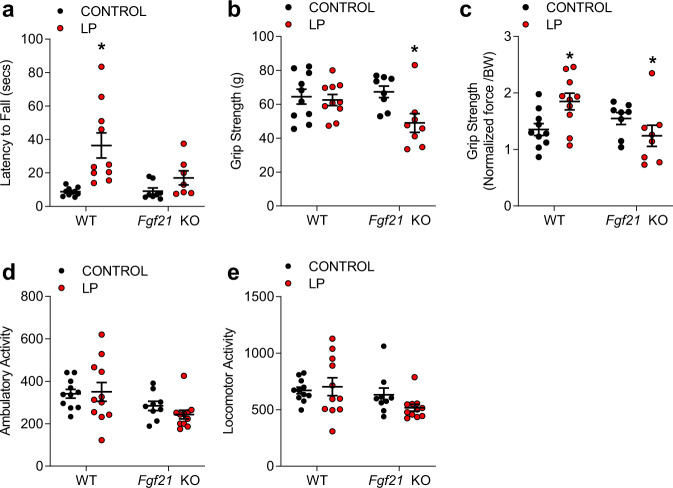
Protein restriction protects against age-related functional decline via FGF21. **a** Latency to fall from an accelerating rod (Rotarod) at 21 months of age in WT and *Fgf21* KO mice on control or LP diets (*n* = 8–12 mice/group; geno*diet *p* = 0.008). **b** Grip Strength at 21 months of age (*n* = 8–12 mice/group; geno*diet *p* = 0.04). **c** Grip Strength normalized to body mass (*n* = 8–12 mice/group; geno*diet *p* = 0.01). **d** Ambulatory activity as measured in the metabolic chambers at 20 months of age (*n* = 8–12 mice/group geno*diet *p* = 0.39). **e** Locomotor activity as measured in the metabolic chambers at 20 months of age (*n* = 8–12 mice/group geno*diet *p* = 0.19). Statistical analyses were conducted using two-way ANOVA. All values are mean ± SEM, with significant posthoc comparison within the diet*genotype interaction noted as *p < 0.05 compared with respective control. Source data are provided as a Source Data file.

Inadequate protein intake, particularly when diagnosed as malnourishment in the elderly, may lead to adverse functional and structural events such as muscle wasting and increase the risk of bone fractures. To assess the contribution of FGF21 to functional performance at 21 months of age, mice fed either control or LP diet were placed on the rotarod to assess physical performance and coordination, which is captured as latency to fall from the rotating rod (Fig. 3a). A significant genotype*diet interaction was observed (*p* < 0.05), with LP diet increasing latency to fall in WT mice (*p* < 0.01), but this effect being largely abolished in *Fgf21 KO* mice (*P* = 0.28). Significant diet*genotype interactions (*p* < 0.01) were also detected for raw grip strength (Fig. 3b) and grip strength normalized to body (Fig. 3c). Raw grip strength was not influenced by the LP diet in WT mice but was significantly increased when normalized to body weight (*p* < 0.05). Similarly, both raw and normalized grip strength was significantly reduced by LP in *Fgf21* KO mice. Finally, ambulatory and locomotor activity within the metabolic chamber at 20 months of age indicated no effect of genotype or diet (Fig. 3d, e). These data indicate that protein restriction increases functional performance, with these beneficial effects being dependent on FGF21.

### FGF21 mediates low protein-induced improvements in glucose homeostasis in aged mice

**Fig. 4:**
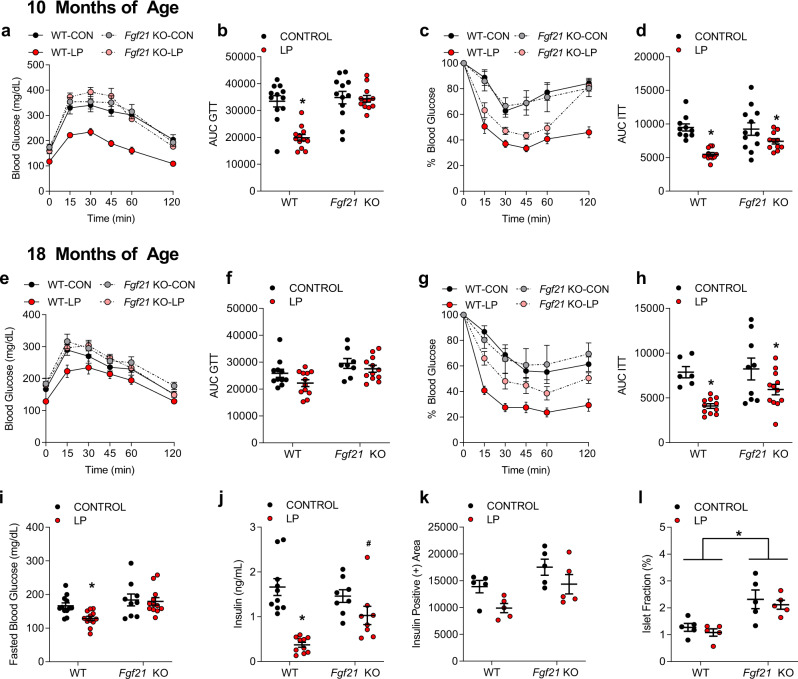
FGF21 mediates low protein-induced improvements in glucose homeostasis in aged mice. **a** Glucose tolerance test measured at 10 months of age in WT and *Fgf21* KO mice control or LP diets (*n* = 8–12 mice/group, group*time *p* = 0.001). **b** Area under the curve glucose as measured in the GTT at 10 months of age (*n* = 8–12 mice/group geno*diet *p* = 0.001). **c** Insulin tolerance test at 10 months of age (*n* = 8–12 mice/group, group*time *p* = 0.001). **d** Area under the curve glucose as measured in the ITT at 10 months of age (*n* = 8–12 mice/group; diet *p* = 0.001). **e** Glucose tolerance test measured at 18 months of age in WT and *Fgf21* KO mice control or LP diets (*n* = 8–12 mice/group group*time *p* = 0.236). **f** Area under the curve glucose as measured in the GTT at 18 months of age (*n* = 8–12 mice/group) geno *p* = 0.004, diet *p* = 0.062. **g** Insulin tolerance test at 18 months of age (*n* = 8–12 mice/group group*time *p* = 0.001). **h** Area under the curve glucose as measured in the ITT at 18 months of age (*n* = 8–12 mice/group; diet *p* = 0.003). **i** Fasting blood glucose at 18 months of age (*n* = 8–12 mice/group; diet *p* = 0.06, geno *p* = 0.0031, geno*diet *p* = 0.128). **j** Serum insulin levels at sacrifice at 22 months of age (*n* = 8–12 mice/group; geno*diet *p* = 0.01). **k** Insulin positive area as measured via insulin IHC on fixed pancreas collected at sacrifice at 22 months of age (*n* = 5 mice/group; geno *p* = 0.001, diet *p* = 0.02). **l** Islet fractional percentage as measured on fixed pancreas collected at sacrifice at 22 months of age (*n* = 5 mice/group; geno *p* = 0.01). Statistical analyses were conducted using one or two-way ANOVA. All values are mean ± SEM, with significant posthoc comparison within the diet*genotype interaction noted as *p < 0.05, ^#^*p* < 0.10 compared with respective control. Source data are provided as a Source Data file.

Low protein fed mice have improved glucose tolerance compared to their control-fed counterparts, furthermore these improvements are correlated with increases in circulating FGF21^18^. To assess the impact of PR induced-FGF21 on glucose homeostasis, i.p. glucose and insulin tolerance tests were conducted at 10 and 18 months of age. Glucose tolerance and insulin sensitivity were significantly improved in WT-LP mice (Fig. 4a–h; *p* < 0.05), as was fasting blood glucose (Fig. 4i; *p* < 0.05). Effects of LP diet on glucose tolerance were completely lost in *Fgf21* KO mice (Fig. 4a, b), but insulin sensitivity was improved in both genotypes (Fig. 4c, g), although the effect was reduced in *Fgf21* KO mice. Serum insulin levels at sacrifice were significantly reduced by LP in WT mice (Fig. 4j, *p* < 0.05), yet this reduction in insulin levels by LP was blunted in *Fgf21* KO mice (*p* = 0.07). Finally, histological analysis of the insulin-positive area and islet fraction of the pancreas at sacrifice indicated a significant main effect of both diet and genotype, but no significant interaction, on insulin-positive area (Fig. 4k; *p* < 0.05), with LP tending to reduce insulin positive area and FGF21 deletion tending to increase it. There was no effect of diet on islet fraction in either group, but islet fraction was significantly higher in *Fgf21* KO mice compared to controls (Fig. 4l; *p* < 0.05). Collectively, these data indicate that the LP diet exerts a persistent effect to protect against age-related declines in glucose homeostasis, and that these diet effects are attenuated in *Fgf21* KO mice.

### FGF21-dependent and independent changes in molecular endpoints in the liver and adipose tissue during protein restriction

**Fig. 5:**
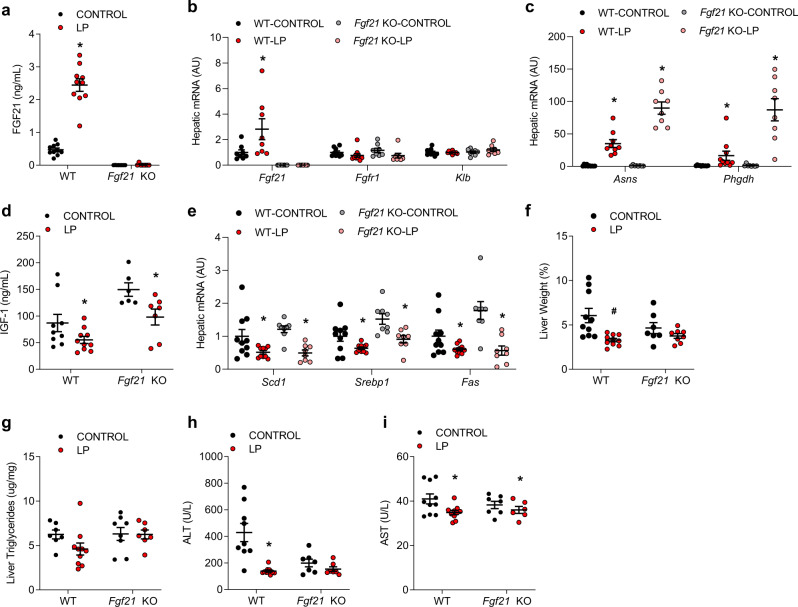
FGF21-dependent and independent changes in molecular endpoints in the liver at 22 months of age. **a** Serum FGF21 levels collected at sacrifice at 22 months of age in WT and Fgf21 KO mice on control or LP diets as in Fig. 2 (*n* = 8–10 mice/group, geno*diet *p* = 0.01). **b** Liver mRNA levels for FGF21 related markers as measured via qPCR at 22 months of age (*n* = 8–10 mice/group, geno*diet *Fgf21* *p* = 0.04, *Fgfr1* p = 0.556, *Klb* = 0.3016). **c** Liver markers of dietary protein restriction (amino acid biosynthetic genes) as measured via qPCR at 22 months of age (*n* = 8–10 mice/group; geno*diet *Asns* *p* = 0.0001, *Phgdh* *p* = 0.001. **d** Serum IGF1 levels at sacrifice at 22 months of age (*n* = 8–10 mice/group; genotype *p* = 0.0004, diet *p* = 0.003, geno*diet *p* = 0.4567). **e** Liver mRNA expression for hepatic lipogenic genes as measured via qPCR at 22 months of age (*n* = 8–10 mice/group; *Scd1* diet *p* = 0.001 geno*diet *p* = 0.553, *Srebp1* diet *p* = 0.001, geno*diet *p* = 0.89, *Fas* diet *p* = 0.001 geno*diet *p* = 0.2395. **f** Liver weight as a percentage of body weight at sacrifice at 22 months of age (*n* = 8–10 mice/group; diet *p* = 0.0026, geno*diet *p* = 0.10). **g** Liver triglyceride content at sacrifice at 22 months of age (*n* = 8–10 mice/group, geno*diet *p* = 0.2271). **h** Serum ALT levels at sacrifice at 22 months of age (*n* = 8–10 mice/group; genotype *p* = 0.01, diet *p* = 0.0003, geno*diet *p* = 0.0057). **i** Serum AST levels at sacrifice at 22 months of age (*n* = 8–10 mice/group; genotype *p* = 0.6662, diet *p* = 0.02, geno*diet *p* = 0.2852). Statistical analyses were conducted using two-way ANOVA. All values are mean ± SEM, with significant main effects of diet or posthoc comparison within the diet*genotype interaction noted as *p < 0.05, ^#^*p* < 0.10 compared with respective control. Source data are provided as a Source Data file.

All mice within this second study were sacrificed at 22 months of age to assess the impact of PR on tissue endpoints. LP induced a robust increase in serum FGF21 in WT mice (Fig. 5a; *p* < 0.01), while FGF21 levels were not detectable in *Fgf21* KO mice. This increase in circulating FGF21 was consistent with a significant increase in liver *Fgf21* mRNA expression (Fig. 5b). The expression of the FGF21 co-receptor *Klb* was not altered in the liver, but *Fgfr1* expression was reduced by the LP diet in both genotypes (*p* < 0.05; Fig. 5b). The amino acid biosynthetic genes *Asns* and *Phgdh* were also significantly increased by LP in both genotypes (Fig. 5c; *p* < 0.05), consistent with continuous protein restriction, and this induction was relatively larger in *Fgf21* KO mice than in WT mice.

Serum IGF-1 levels (Fig. 5d) were independently increased by the deletion of FGF21 (genotype *p* = 0.004) and reduced by LP diet (diet *p* = 0.003); however, the diet*genotype interaction was not statistically significant. LP diet reduced the expression of liver genes associated with lipogenesis (*Scd-1, Srebp1c, Fas*) in both genotypes (diet *p* < 0.05, genotype and G*D = NS; Fig. 5e). Liver mass was significantly reduced by diet (*p* = 0.002; Fig. 5f), and there was a trend (*p* = 0.10) towards a diet*genotype interaction with WT mice exhibiting a larger reduction compared to *Fgf21* KO mice. Liver triglyceride content displayed a similar pattern; however, differences were not statistically significant (Fig. 5g). Finally, changes in circulating ALT and AST levels were used as markers of liver health (Fig. 5h, i). A significant diet*genotype interaction was observed for ALT (*p* = 0.0057), with the LP diet markedly reducing ALT levels in WT-LP mice but not in *Fgf21* KO-LP mice. However, this difference is driven largely by differences in baseline ALT between WT-CON and *Fgf21* KO-CON mice. Conversely, AST levels were modestly reduced by diet in both genotypes (diet *p* = 0.02), but the diet*genotype interaction was not statistically significant (*p* = 0.285).

**Fig. 6:**
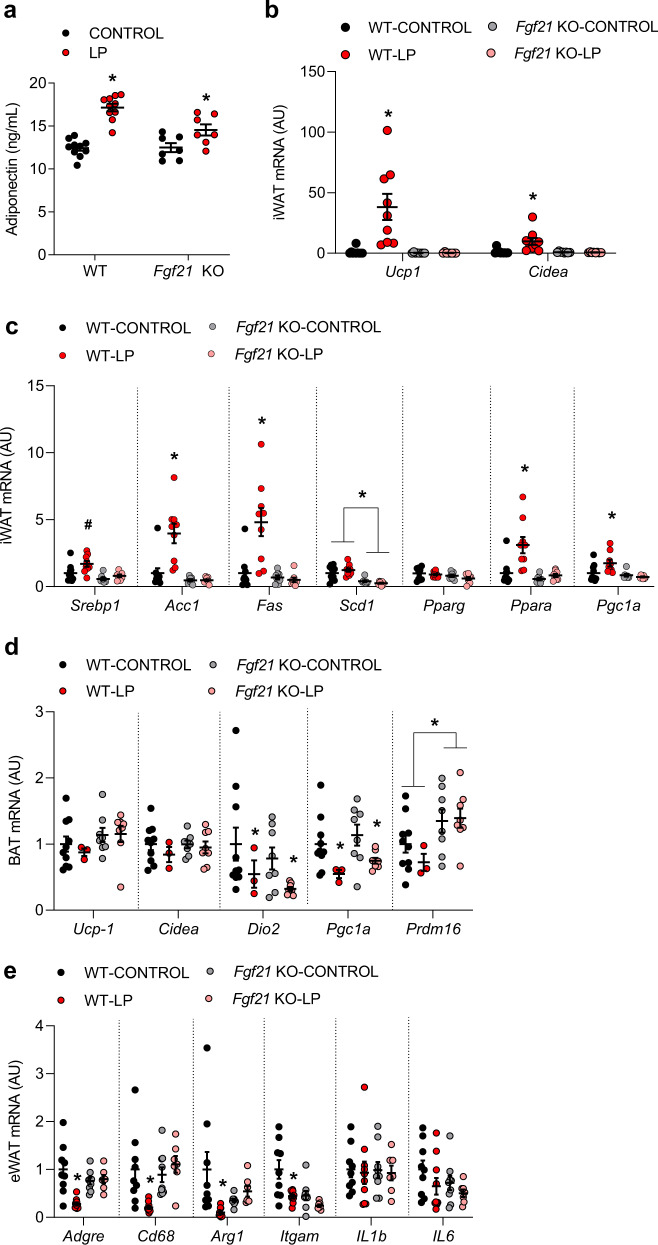
FGF21-dependent and independent changes in molecular endpoints in the adipose tissue at 22 months of age. **a** Serum adiponectin collected at 22 months of age (*n* = 8–10 mice/group; geno*diet *p* = 0.009). **b** Thermogenic gene expression in inguinal white adipose tissue (iWAT) collected at sacrifice at 22 months of age (*n* = 8–10 mice/group; geno*diet *Ucp1* *p* = 0.0023, *Cidea* *p* = 0.0065). **c** Energy metabolism gene expression in iWAT at 22 months of age (*n* = 8–10 mice/group; *Srebp1* diet *p* = 0.01 geno*diet *p* = 0.1876, *Acc1* geno*diet *p* = 0.002, *Fas* geno*diet *p* = 0.002, *Scd1* geno*diet *p* = 0.1388, *Pparg* geno*diet *p* = 0.6344, *Ppara* geno*diet *p* = 0.01, *Pgc1a* geno*diet *p* = 0.02). **d** Expression of thermogenic markers within BAT (*n* = 3–10 mice/group; *Ucp1* geno*diet *p* = 0.6124, *Cidea* geno*diet *p* = 0.5739, *Dio2* diet *p* = 0.05 geno*diet *p* = 0.99, *Pgc1a* diet *p* = 0.007 geno*diet *p* = 0.37, *Prdm16* geno *p* = 0.0079 geno*diet *p* = 0.37). **e** Expression of inflammatory markers within visceral adipose tissue (*n* = 8–10 mice/group; *Adgre* geno*diet *p* = 0.009, *Cd68* *p* = 0.006, *Arg1* geno*diet *p* = 0.01, *Itgam* diet *p* = 0.002 geno *p* = 0.03 geno*diet *p* = 0.14, *IL1b* geno*diet *p* = 0.9, *IL6* geno*diet *p* = 0.67). Statistical analyses were conducted using two-way ANOVA. All values are mean ± SEM, with significant main effects of diet or posthoc comparison within the diet*genotype interaction noted as *p < 0.05, ^#^*p* < 0.10 compared with respective control. Source data are provided as a Source Data file.

Adipose tissue-related markers were also altered by LP diet at 22 months of age. Circulating adiponectin was significantly increased by LP diet in both WT and *Fgf21* KO mice (Fig. 6a). Conversely, the LP diet robustly increased thermogenic regulatory genes (*Ucp1*, *Cidea*; Fig. 6b, *p* < 0.01) within inguinal white adipose tissue (iWAT) in WT mice, but this effect was lost in *Fgf21* KO mice. LP diet also induced genes associated with adipose tissue lipid oxidation and metabolic function, including *Acc1, Fas, Ppara*, and *Pgc1a* (Fig. 6c; *p* < 0.05), and again these effects were lost in *Fgf21* KO mice. Interestingly, despite the increase in EE and induction of thermogenic genes in iWAT, we observed no increase in thermogenic markers within the BAT of either WT or *Fgf21* KO mice on LP (Fig. 6d). Finally, LP diet reduced the expression of multiple inflammatory markers within epididymal white adipose tissue, most notably *Adgre*, *Cd68*, *Arg1*, and *Itgam* (Fig. 6e), although neither *IL1b* nor *IL6* expression was altered. This effect of LP was largely blocked in *Fgf21* KO mice, although baseline levels of *Arg1* and *Itgam* were already low in *Fgf21* KO-CON mice. Collectively, these data provide evidence that protein restriction alters multiple metabolic targets within adipose tissue, and that most but not all of these effects are dependent on FGF21.

### Protein restriction protects against the detriments of diet-induced obesity during middle age

**Fig. 7:**
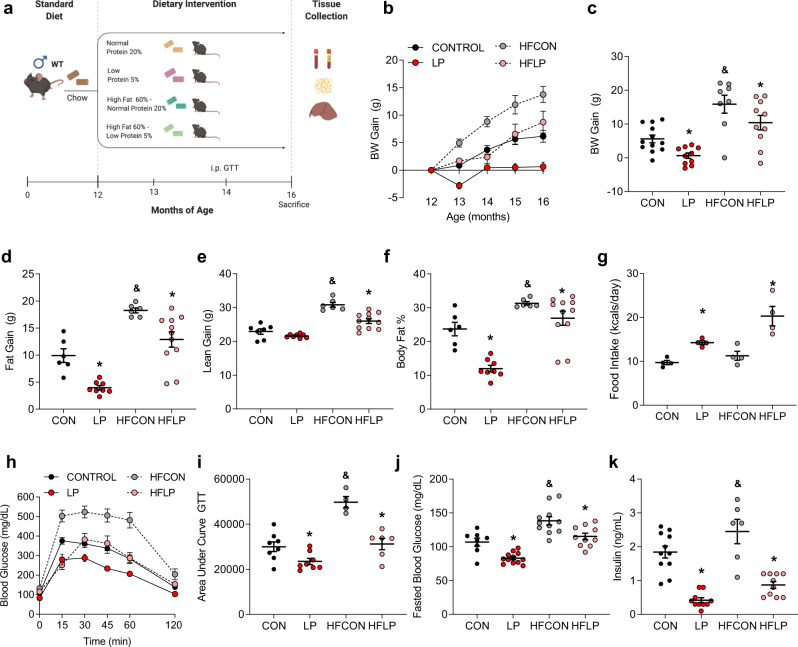
Protein restriction promotes beneficial metabolic effects and protects against diet-induced obesity when initiated at middle age. **a** Schematic representation of middle-aged study. Wildtype and *Fgf21*-KO mice were placed on CON, LP, HFCON, or HFLP diet at 12 months of age, with various metabolic endpoints assessed throughout the study as indicated and tissue collection at 16 months of age. **b** Body weight gain from initiation of diets (*n* = 12 mice/group). **c** Body weight gain from diet initiation to sacrifice at 16 months of age (*n* = 8–12 mice/group; fat *p* < 0.0001, protein *p* = 0.0046, fat*protein *p* = 0.8814). **d** Body fat gain from diet initiation to sacrifice at 16 months of age, as measured by NMR (*n* = 6–12 mice/group; fat *p* < 0.0001, protein *p* < 0.0001, fat*protein *p* = 0.816). **e** Body lean gain from diet initiation to sacrifice at 16 months of age, as measured by NMR (*n* = 6–12 mice/group; fat *p* = 0.001, protein *p* = 0.001, fat*protein *p* = 0.0144. **f** Percent body fat at sacrifice at 16 months of age (*n* = 6–12 mice/group; fat *p* < 0.0001, protein *p* < 0.0001, fat*protein *p* = 0.218). **g** Average daily food intake throughout the experiment (*n* = 4 cages/group fat *p* = 0.6676, protein *p* = 0.0001, fat*protein *p* = 0.285). **h** Glucose tolerance test conducted at 14 months of age (2 months on diet (*n* = 6–8 mice/group; group*time *p* = 0.001). **i** Area under the curve glucose for the GTT at 14 months of age (*n* = 6–8 mice/group fat*protein *p* = 0.01). **j** Fasting blood glucose at 14 months of age (*n* = 6–8 mice/group; fat *p* = 0.0001, protein *p* = 0.0001, fat*protein *p* = 0.94). **k** Serum insulin levels at sacrifice at 14 months of age (*n* = 6–8 mice/group; fat *p* = 0.0033, protein *p* = 0.0001, fat*protein *p* = 0.4746). Statistical analyses were conducted using two-way ANOVA with dietary fat content and dietary protein content as main effects. All values are mean ± SEM, with significant main effects of protein or posthoc comparisons within the fat*protein interaction noted as *p < 0.05 vs respective control, and main effects of fat noted as ^&^*p* < 0.05 HFCON vs CON. Source data are provided as a Source Data file.

To further expound on the efficacy of PR to reduce the progression of age-related metabolic decline, forty-eight 12-month-old male C576BL/6 J mice were randomly assigned to one of 4 diets (12 mice/diet): control (CON) and low protein (LP) diets as above, as well as two additional diets, HFCON and HFLP, that varied protein on the background of a 60% high-fat diet. Mice consumed these diets ad libitum from 12 to 16 months of age (Fig. 7a) to test the impact of protein restriction in middle-aged mice with diet-induced obesity. As expected, HFCON mice gained weight more rapidly than CON mice (Fig. 7b, c; *p* < 0.05), and both LP and HFLP reduced weight relative to their controls. HFCON gained both fat and lean mass at a greater rate than any group, and both LP and HFLP mice exhibit reduced fat and lean gain relative to their controls (Fig. 6d, e; *p* < 0.05). However, the reduction in fat gain was larger than lean gain, such that LP and HFLP show a decrease in body adiposity (Fig. 7f). These changes in weight and body composition were associated with increases in food intake in both LP and HFLP relative to their controls (Fig. 7g; *p* < 0.05). Consistent with changes in body composition, HFCON mice exhibit impaired glucose tolerance (Fig. 7h, i; *p* < 0.05), increased fasting blood glucose (Fig. 7j; *p* < 0.05), and increased serum insulin (Fig. 7k; *p* < 0.05) as compared to CON mice. Both LP and HFLP improved glucose tolerance and lowered fasting glucose and insulin levels (*p* < 0.05). Strikingly, HFLP normalized body adiposity, glucose tolerance, and fasting glucose and insulin levels, even though these middle-aged animals remained on a 60% high-fat diet.

### Protein restriction alters liver and adipose tissue endpoints in middle-aged mice

**Fig. 8:**
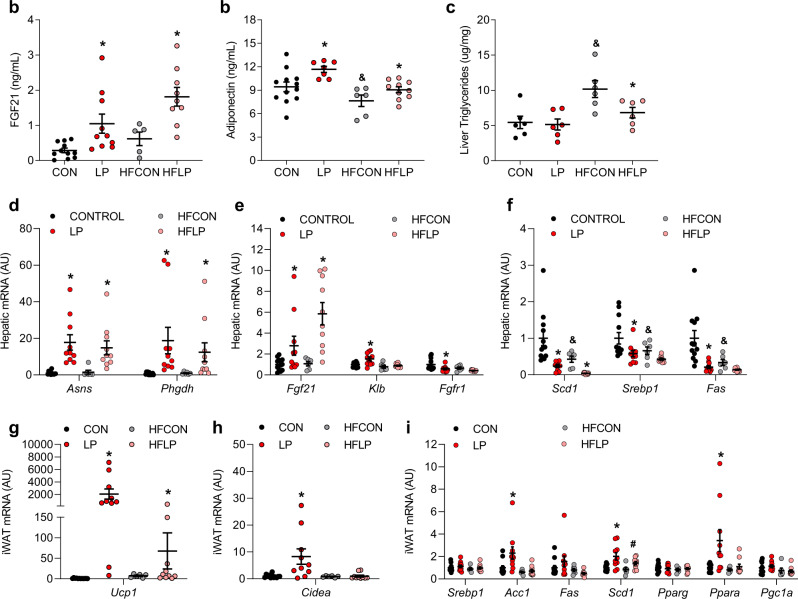
Serum, liver, and adipose tissue endpoints following the initiation of dietary protein restriction in middle age. **a** Serum FGF21 concentrations in wildtype and *Fgf21*-KO mice consuming control or LP diet from 12 to 16 months of age as Fig. 7a (*n* = 6–10 mice/group; protein *p* = 0.0002). **b** Serum Adiponectin concentrations at 16 months of age (*n* = 6–10 mice/group; fat *p* = 0.0007, protein *p* = 0.004). **c** Liver triglyceride content at 16 months of age (*n* = 6–10 mice/group; fat *p* = 0.0023, protein *p* = 0.06, fat*protein *p* = 0.11). **d** Liver amino acid biosynthetic gene expression (markers of dietary protein restriction) as measured via qPCR at 16 months of age (*n* = 6–10 mice/group; *Asns* protein *p* = 0.003, *Phgdh* protein *p* = 0.02). **e** Liver mRNA expression of FGF21 signaling genes as measured via qPCR at 16 months of age (*n* = 6–10 mice/group; *Fgf21* protein *p* = 0.01, *Klb* fat *p* = 0.001, protein *p* = 0.01, fat*protein *p* = 0.08, *Fgfr1* fat *p* = 0.04, protein *p* = 0.01) **f** Liver mRNA expression of lipogenic genes as measured via qPCR at 16 months of age (*n* = 6–10 mice/group, *Scd1* fat *p* = 0.008, protein *p* = 0.0002, *Srebp1* fat *p* = 0.04, protein *p* = 0.009, *Fas* fat *p* = 0.01, protein *p* = 0.001). **g** iWAT expression of *Ucp1* at 16 months of age (*n* = 6–10 mice/group; fat*protein *p* = 0.02). **h** iWAT expression of *Cidea* at 16 months of age (*n* = 6–10 mice/group; fat*protein *p* = 0.03). **i** iWAT expression of metabolic genes at 16 months of age (*n* = 6–10 mice/group; *Srebp1* *p* = NS, Acc1 fat*protein *p* = 0.08, *Fas* fat *p* = 0.04, *Scd1* fat *p* = 0.08, protein *p* = 0.008, *Pparg* *p* = NS, *Ppara* fat*protein *p* = 0.06, *Pgc1a* fat *p* = 0.03). Statistical analyses were conducted using two-way ANOVA. All values are mean ± SEM, with significant main effects of protein or posthoc comparison within the fat*protein interaction noted as *p < 0.05 vs respective control and main effects of fat noted as ^&^*p* < 0.05 HFCON vs CON. Source data are provided as a Source Data file.

Lastly, we addressed the effect of age on a subset of metabolic genes in liver and adipose tissue during PR. As expected, LP and HFLP significantly increased circulating FGF21 (Fig. 8a; *p* < 0.05) and adiponectin (Fig. 8b; *p* < 0.05). Within the liver, HFCON increased liver triglyceride content relative to control, and this effect was prevented by HFLP (Fig. 8c). Both LP and HFLP increased liver amino acid biosynthetic genes (*Asns* and *Phgdh*; Fig. 8d), as well as hepatic *Fgf21* expression (Fig. 8e). Both high fat and low protein independently reduced liver lipogenic gene expression (Fig. 7f). LP diet induced a strong increase of thermogenic gene expression (*Ucp1*, *Cidea*) in iWAT (Fig. 8g, h; *p* < 0.05). The increase in *Ucp1* expression was also observed but smaller in magnitude in HFLP fed mice, while there was no increase in *Cidea* expression in HFLP. This pattern of a blunted effect on adipose tissue gene expression was also observed for related genes, with the LP-induced increase of *Acc1*, *Fas*, and *Ppara* being lost in HFLP (Fig. 8i).

While obesity may occur at any age, the detrimental effects of obesity are magnified with advancing age. These data suggest that despite continued exposure to a 60% high fat diet at middle-age, the HFLP diet was sufficient to protect against the negative effects of high fat diet-induced obesity.

## Discussion

The ability of PR to reduce the physical signs of aging in model organisms has been recognized for over 75 years^54–56^, with more recent work demonstrating the beneficial effects of dietary protein or amino acid restriction in a variety of species^10–15^. The specific mechanisms driving the anti-aging effects of protein restriction are not well known, and the primary research focus has been various cell-autonomous nutrient-sensing pathways, such as mTOR, AMPK, sirtuins, or GCN2^12,32–39^. However, our prior work in young mice indicated that the endocrine hormone FGF21 was required for young mice to detect and metabolically adapt to a low protein diet^42,45^. Importantly, FGF21 has been previously linked to lifespan extension, as transgenic overexpression of FGF21 increases lifespan and inhibits growth in mice^52,57^. However, no study has previously tested whether FGF21 contributes to lifespan extension in a dietary or otherwise physiological model. To test whether FGF21 was required for the longevity effects of PR, male control and *Fgf21*-KO mice were placed on a low protein diet at 3 months of age and were then allowed to age naturally. Collectively, the data demonstrate that FGF21 is not only required for the longevity effects of PR, but the absence of FGF21 converts the beneficial effect of PR to a detrimental effect. This work provides clear evidence that FGF21 plays a physiological role in longevity and identifies a unique mechanism that explains PR-induced longevity in a mammalian model.

The differential effects of PR on lifespan in the WT and *Fgf21* KO mice were mirrored by changes in a variety of physiological and metabolic markers, both within the lifespan group and in a second experimental cohort (termed metabolic group) of WT and *Fgf21* KO mice that were fed either control or LP diets from 3 to 22 months of age. Consistent with prior work in young mice, the LP induced a persistent reduction in body weight gain over the first 2 years of life in WT-LP mice. However, we do not interpret these reductions in growth as evidence of malnutrition or poor health. Instead, the WT-LP mice in the lifespan group exhibited a reduction in clinical markers of frailty and subjectively looked healthier and more robust compared to their controls. Similarly, WT-LP mice in the metabolic group exhibited reduced total adiposity, and measures of physical performance (rotorod, grip strength) were either unchanged or improved by PR in WT mice, although we acknowledge that these effects are likely to be driven by differences in body weight in WT mice. While LP diet did reduce total BW in WT mice, there was no evidence that prolonged PR negatively impacted physical function. Instead, we suggest that the WT-LP mice were smaller but healthier, consistent with the effects on lifespan, and not unlike mice on chronic CR. However, unlike CR, these mice were consuming food ad libitum with no additional intervention on the part of the experimenter.

While long-term PR led to beneficial effects on frailty and physical performance in WT mice, the *Fgf21* KO mice exhibited a much more complex response. Most notably, the *Fgf21* KO mice were initially unresponsive to the diet, supporting our prior observation that FGF21 is required for mice to physiologically adapt to a state of protein restriction. While this lack of adaptation appears benign in young mice, over time the LP diet exerted increasingly negative effects in *Fgf21* KO mice. For example, *Fgf21* KO-LP mice exhibit age-related weight loss earlier than any other group, several clinical markers of aging and frailty (alopecia, coat condition, kyphosis) were increased by LP in the *Fgf21* KO mice, the subjective appearance of *Fgf21* KO-LP mice declined earlier than any other group, *Fgf21* KO-LP mice showed no improvement in rotarod performance, and finally grip strength was reduced. Interestingly, separate work specifically focusing on the kidney in these same animals observed increased kidney inflammation in *Fgf21* KO-LP mice, again suggesting that protein restriction induces negative outcomes in the absence of FGF21^58^. Collectively, these data paint a consistent picture in which protein restriction improves functional performance and reduces frailty in WT mice, but conversely leads to long-term negative outcomes for performance and frailty in *Fgf21* KO mice.

The metabolic cohort also allowed us to directly assess metabolic and tissue endpoints in WT and *Fgf21* KO mice following long-term protein restriction. Many of these effects are consistent with our prior work in young mice. In WT mice the LP diet persistently increased both food intake and energy expenditure, while changes in food intake and energy expenditure were absent in *Fgf21* KO mice even at 22 months of age. Thus the effects of the LP diet seem to be consistent throughout the lifespan, and also to consistently require FGF21. Dietary PR has also been shown to improve measures of metabolic health in young mice^3,43,45^, and in WT mice the LP diet enhanced glucose tolerance and insulin sensitivity and reduced fasting blood glucose and insulin, with these effects being largely absent in *Fgf21* KO mice. It seems likely that these effects are driven by LP-induced reductions in body weight and adiposity, as liver weight and triglyceride content were also reduced in WT but not *Fgf21* KO-LP. However, we point out prior work indicating that PR engages unique signaling mechanisms that are not engaged by an equivalent weight loss induced by general food restriction, most notably the increase in FGF21^45^.

PR also altered the expression of multiple metabolic genes within the liver and adipose tissue at 22 months of age, including a consistent and robust increase in both circulating FGF21 protein and liver *Fgf21* mRNA expression, which suggest that PR persistently increases FGF21 for the full duration of dietary exposure. Interestingly, the LP-induced change in the expression of several liver genes was intact in *Fgf21* KO mice. Our earlier work indicated that the LP-induced reductions in the expression of liver lipogenic genes (*Scd1,Srebp1,Fas*) were lost in *Fgf21* KO mice^41^, but more recent work^44^ and this study indicate that the reduction in lipogenic genes is intact LP-fed *Fgf21* KO mice. Interestingly, the LP diet also significantly reduced circulating IGF-1 levels in both control and *Fgf21* KO mice, indicating that this well-known effect of PR is not FGF21-dependent. However, interpretation of these data is complicated by the fact that the deletion of FGF21 itself had an independent effect to increase IGF-1, such that *Fgf21* KO mice had increased IGF-1 levels on both diets. The role that IGF-1 might play in the metabolic or lifespan effects we observe is currently not clear, but these changes in IGF-1 are consistent with prior work linking FGF21 to hepatic GH signaling and the actions of mTOR^9,57,59–61^. Circulating adiponectin levels were also increased by PR, and this effect was retained in *Fgf21* KO mice. Within adipose tissue, our work and that of others indicate that protein restriction promotes a remodeling (beiging) of inguinal white adipose tissue^44,45^, and in these aged mice we observed a similar, FGF21-dependent induction of thermogenic gene expression. LP diet also altered the expression of genes related to lipid metabolism (*Fas, Acc1, Ppara*), with these effects being dependent on FGF21. Interestingly, despite the increase in whole-body EE and the remodeling of iWAT, we did not observe increased thermogenic markers within BAT. These data are consistent with prior work in our lab in which BAT *Ucp1* mRNA expression was increased after 2 weeks of LP exposure, but not after 27 weeks^41^. However, these assessments depend exclusively on measures of mRNA expression which do not necessarily reflect the total thermogenic capacity of BAT^62^, and it is therefore problematic to conclude that BAT may be uninvolved in the changes in whole body energy expenditure. Finally, consistent with changes in whole-body adiposity and metabolic health, the LP diet also reduced various markers of inflammation, particularly macrophage markers, within visceral adipose tissue of WT mice, and these effects were largely blunted in *Fgf21* KO mice. In summary, these data indicate that changes in whole-body metabolism are also linked to specific adaptations within peripheral tissues, particularly liver and adipose tissue. While changes in adipose tissue appear to be largely downstream of and dependent on increases in FGF21, the liver’s response to PR, particularly in terms of changes in gene expression, were largely intact in *Fgf21* KO mice and thus appear to be independent of FGF21. These observations are thus consistent with a model in which the liver initially detects PR and increases circulating FGF21 as an endocrine signal, with FGF21 acting in a variety of sites, most notably the brain, to coordinate adaptive metabolic responses^40,45^.

While the data described above clearly indicate that PR, via the induction of FGF21, protects against age-related metabolic decline, these data are based on a model in which PR is initiated in young adult mice (3 months of age). To test whether PR would also improve metabolic health in middle-aged animals, 12-month-old WT mice were placed on the previously described control and LP diets, as well as two additional diets that contained 60% fat. These latter diets were designed to test whether PR is sufficient to prevent the acceleration of age-associated metabolic dysfunction that occurs with diet-induced obesity. Collectively, these data demonstrate that the effects of protein restriction were retained in middle-aged mice, with LP reducing weight, BW gain, and adiposity, improving glucose homeostasis, and altering liver and adipose tissue gene expression when the diet was initiated at 12 months. Most notably, while HFCON diet exacerbated age-associated metabolic dysfunction as expected, these negative effects were largely prevented by HFLP. It is quite compelling to observe that mice on HFLP were generally similar to control-fed mice despite consuming a 60% fat diet ad libitum. These data are similar to a recent publication indicating that PR initiated at 18 months of age increases energy expenditure and adipose browning, although no effects on glucose homeostasis were observed^63^. Taken together, these data provide strong evidence that PR improves metabolic health when initiated in middle age, and importantly that PR can prevent the negative effects of high-fat diet exposure in this model.

Our data strongly suggest that PR improves healthspan and longevity via FGF21, yet it is important to comment on the limitations within our study. First, genetic variation has been shown to improve or blunt the metabolic benefits of diet on healthspan or lifespan^64^, and this variability in genetic response could extend to PR. Testing the effects of PR in multiple strains or mixed background strains such as HET-3 or the Diversity Outbred^65–68^ may provide evidence that is more translatable to humans. Although all mice in the current study are on the B6 background, it should be noted that the WT and *Fgf21* KO mice are derived from separate colonies (not littermates), with potentially subtle differences in genetic background. Most notably, *Fgf21* KO on the control diet exhibit reduced overall body weight and increased median lifespan compared to WT mice, and the mechanism underlying this difference is currently unclear. Second, sex also alters the response to healthy diets^69^. The beneficial effects of PR have been primarily illustrated in males, and only males were used in the current study. Therefore, the extent of sexual dimorphism in the improvements in healthspan and lifespan with PR remains unclear. Notably, a recent study reported that PR only improves metabolic health in males and ovariectomized female mice, suggesting that the benefits of PR are blunted in female mice^70^. New data indicate that neither PR nor BCAA restriction improved lifespan in females compared to males and that the benefits of BCAA restriction were reduced in middle-aged males^31^. On the other hand, initial findings in female rats showed that PR extended lifespan when initiated 120d of age^71^. Finally, this study used only a single diet to model protein restriction (5% casein) and a single genetic model to delete FGF21 (*Fgf21* KO mice). It is possible that the observed effects, including the contribution of FGF21, might vary with differing levels of protein content and uniquely interact with differences in sex or genetic background.

In summary, our work collectively supports multiple overarching conclusions. First, in male C57BL/6 J mice the restriction of dietary protein intake exerts beneficial effects on body weight gain, adiposity, glucose homeostasis, physical performance, and metabolic health, with these effects ultimately reducing frailty and increasing lifespan. Second, these beneficial effects can be reproduced if the diet is initiated in middle age, where PR also protects against the harmful effects of diet-induced obesity during aging. Finally, and most importantly, nearly all these PR-induced beneficial effects depend on the liver-derived hormone FGF21. The inability to detect protein restriction in *Fgf21*-KO mice is initially benign but ultimately leads to negative outcomes in late life, such that *Fgf21*-KO mice on LP die earlier than their controls. Collectively, these data demonstrate that increases in circulating FGF21 mediate the extension of lifespan and improvement in healthspan during dietary protein restriction, and thereby identify a novel mechanism that drives the increase in longevity in response to dietary protein restriction in mammals.

## Methods

### Animals and diets

All procedures involving animals were approved by the PBRC Institutional Animal Care and Use Committee and were performed following the guidelines and regulations of the NIH Office of Laboratory Animal Welfare. Male C57BL/6 mice (WT, Jackson Lab) were used in all studies. *Fgf21*-deficient mice on the C57BL/6 background (*Fgf21*-KO) were originally provided by Steven Kliewer (University of Texas Southwestern, Dallas, Texas, USA) and maintained as a colony at PBRC. Diets were formulated and produced by Research Diets as previously described^42,45^ and were designed to be isocaloric by equally varying protein and carbohydrate while keeping fat constant. Control diets (CON) contained 20% casein (by weight) as the protein source, while the low protein diet (LP) contained 5% casein. High-fat diets (HF) also contained 20% casein (HFCON) and 5% casein (HFLP), respectively, but on a background of 60% fat. All diet compositions are provided in Table S1. At the end of the study mice were sacrificed during the mid-light cycle in the fed state (unless otherwise noted) using acute exposure to CO_2_ followed by rapid decapitation. Trunk blood was also collected at sacrifice, allowed to clot overnight at 4 °C, centrifuged at 3000xg, and serum collected. Tissues were collected and snap-frozen in liquid nitrogen for further analysis.

### Experimental design

To link dietary protein restriction-induced FGF21 signaling to lifespan, male wildtype and *Fgf21-*KO mice were entered into the study at approximately 3 months of age, group-housed (4 per cage) at room temperature (23 °C), and placed on control or LP diet *ad libitum* (30 mice/diet/genotype). Mice were maintained on these diets until natural death or clinically determined as failure to thrive, with failure to thrive being jointly determined by laboratory staff and veterinary care staff and triggering humane euthanasia. The mice in this longevity study were solely utilized to examine the effects of dietary protein content on lifespan and determine if FGF21 is required. Therefore, this group was minimally handled except for routine body weight measurements and a one-time frailty index assessment at 18 months of age. The experimental timeline is presented in Fig. 1A (Schematics were created with BioRender).

To test if FGF21 is required for the effects of dietary protein restriction on metabolic health during aging, male wildtype and *Fgf21-*KO mice were entered into the study at approximately 3 months of age and group-housed (4 per cage) at room temperature (23 °C). Mice were placed on either control or LP diet *ad libitum* until 22 months of age (12 mice/diet/genotype). Mice that were clinically determined as being unable to thrive were removed from the study and euthanized as above. Bodyweight and food intake were recorded weekly throughout the experiment. Body composition was measured via TD-NMR (Bruker Minispec) at the start (3 months of age), middle (12 months of age), and end (22 months of age) of the study. Glucose homeostasis was assessed by glucose tolerance test and intraperitoneal insulin tolerance test at 10 and 18 months of age. Energy expenditure, ambulatory activity, and locomotor activity were measured at 12 and 20 months of age in metabolic chambers (23 °C) via TSE Systems (Phenomaster / Labmaster). Assessment of motor coordination via RotaRod treadmill (Med Associates) and grip strength (Harvard Apparatus) was measured at 21 months of age. Mice were sacrificed at 22 months of age for blood and tissue collection for further processing and analysis. The experimental timeline is presented in Fig. 2A (Schematics were created with BioRender).

To examine if age impacts the effect of dietary protein restriction to improve metabolic health, 12 month-old male mice were randomly assigned to one of 4 diets with 12 mice/diet: control (CON), LP, HFCON, or HFLP diet *ad libitum*. All mice were initially adapted to the control diet and then transitioned to their experimental diet while in the metabolic chambers (TSE Systems; PhenoMaster). After 14 days, mice were transitioned to standard cages, group-housed 4 per cage, and maintained on diet for four months. Bodyweight and food intake were recorded weekly throughout the experiment. Body composition was measured via TD-NMR (Bruker Minispec) at the start (12 months of age) and the end (16 months of age) of experimental diets. Glucose homeostasis was accessed by glucose tolerance test at 14 months of age. Mice were sacrificed at 16 months of age for blood and tissue collection for further processing and analysis. The experimental timeline is presented in Fig. 6A (Schematics were created with BioRender).

### Frailty

Frailty was assessed in mice by using 5 clinically observed, noninvasive, health-related deficits based on the Howlett Frailty Index^72,73^. The deficits were scored as either absent (score 0), mild (score 0.5), or severe (score 1). Two independent, blinded individuals scored each mouse, with these numbers averaged before final analysis.

### Grip strength

Forelimb grip strength (Harvard Apparatus) was evaluated at 21 months of age. The grip strength meter was positioned horizontally, and the mouse was held by the tail and lowered towards the mesh grid such that the forelimbs were allowed to grasp the metal grid. The mouse was then pulled backward in a horizontal fashion with grip strength measured as peak tension in grams. The measurement was performed three times per mouse and the scores were averaged for analysis.

### Motor coordination

Rotarod (Med‐Associates, St Albans, VT) was used to assess motor function and coordination at 21 months of age. Mice were placed on a slowly accelerating rod that progresses over 300 sec, and the latency to fall was recorded for each animal. Mice were measured in a single day, with each mouse tested over 3 trials with an inter‐trial interval of 30‐40 min. Mean latency to fall was calculated per mouse and used for statistical analysis.

### Glucose tolerance test (GTT)

Sixteen-hour-fasted mice underwent GTT by i.p. injection with 2 g glucose per kg of BW. Blood glucose levels were measured at 0, 15, 30, 45, 60, and 120 min via a handheld glucometer (Accu Check; Roche Diabetes Care, Inc. Indianapolis IN). The data for GTT are represented as mg/dL and as the area under the curve (AUC). Fasted glucose levels are reported at the time of GTT.

### Insulin tolerance test (ITT)

Three-hour-fasted mice were injected i.p. with 0.75 IU human insulin (Eli Lilly and Company, Indianapolis, IN) per kg of BW. Blood glucose levels were measured at 0, 15, 30, 60, and 120 min with a glucometer (Accu Check; Roche Diabetes Care, Inc. Indianapolis IN). The ITT data are presented as a percentage of baseline glucose and as the area under the curve (AUC).

### Real-time PCR

RNA extraction and real-time PCR were conducted as described previously^36,42^. Total RNA was extracted from liver and inguinal white adipose tissue (iWAT) using TRIzol reagent following the manufacturer’s protocol (Invitrogen), with the addition of an RNeasy Lipid Tissue Mini Kit (QIAGEN) for iWAT. RNA purity and quantity were determined by spectrophotometry using a NanoDrop (Thermo Scientific). cDNA synthesis was performed with iScript (BioRad) and mRNA was quantified on the ABI 7900 platform using the ABI SYBR Green PCR Master Mix in optical 384-well plates (Applied Biosystems). Primer pairs were designed using the IDT RealTime qPCR Primer Design tool to span an intro-exon boundary (Table S3). Target gene expression was normalized with cyclophilin as the endogenous control.

### Immunoassay determination of FGF21, insulin, adiponectin, and IGF-1

Serum concentrations of FGF21 (no. RD291108200R, Mouse and Rat FGF-21 ELISA, BioVendor), insulin (#EZRMI-13K, Rat/Mouse Insulin ELISA, EMD Millipore Corporation), adiponectin (#EZMADP-60K, Mouse Adiponectin EMD Millipore Corporation), and IGF-1 (ab100695, Mouse IGF1 ELISA, Abcam) were determined via ELISA according to the manufacturer’s recommended protocol.

### Colorimetric determination of hepatic triglycerides and serum ALT and AST

Concentrations of hepatic triglycerides were measured in hepatic homogenates with a colorimetric assay (no. 10010303, Cayman Chemical) according to the protocol recommended by the manufacturer

Concentrations of ALT (MAK052-Sigma Aldrich, Alanine Aminotransferase Activity Assay Kit) and AST (MAK055-Sigma Aldrich, Aspartate Aminotransferase Activity Assay Kit) in serum were determined with a colorimetric coupled enzymatic assay according to the procedure recommended by the manufacturer

### Pancreas histology and immunohistochemistry

After fixation in 10% neutral buffered formalin (NBF) for 24–48 hr, the pancreatic tissue was embedded in paraffin. Five μm sections were cut onto positively charged slides and detection of insulin was performed on a Leica Bond-Max (Leica Biosystems, Melbourne, Australia) using the Bond Polymer Refine detection kit. The insulin primary antibody was guinea pig anti-insulin (1:800, #18-0067, Invitrogen, Grand Island, NY) with secondary detection performed using HRP-conjugated rabbit anti-guinea pig (1:800, A5545, Sigma, Saint Louis, MO). Stained sections were imaged using a Hamamatsu NanoZoomer digital slide scanner at 20x resolution. For determination of insulin positive area, insulin positive islets were detected and quantified using a custom application that detects insulin for each islet present within a section using the Visiopharm VIS software version 5.0.5.

### Quantification and statistical analysis

Data were analyzed using the SAS version 9 or JMP Pro version 16 software packages (SAS Institute) using one-way, two-way, or repeated-measures ANOVA using the general linear model procedure. When experiment-wide tests were significant, post hoc comparisons were made using the LSMEANS statement with the PDIFF option, and represent least significant differences tests for pre-planned comparisons, generally within the diet*genotype or fat*protein interaction. Average daily energy expenditure was analyzed via analysis of covariance (ANCOVA) with body weight as the covariate using the general linear model procedure of SAS V9. Kaplan-Meier survival and Cox Proportional Hazards analysis were conducted using the JMP Pro 16 software program. All data are expressed as mean ± SEM, with a probability value of 0.05 considered statistically significant.

### Reporting summary

Further information on research design is available in the Nature Research Reporting Summary linked to this article.

## Supplementary information

Supplementary Information

Reporting Summary

## Data Availability

All data generated in this study are provided in the Supplementary Information/Source Data file, and from the corresponding author upon reasonable request. Source data are provided with this paper.

## Source data

Source Data
