# Supplementary material for: FGF21 is required for protein restriction to extend lifespan and improve metabolic health in male mice
Source: Nat Commun. 2022 Apr 7;13:1897. doi: 10.1038/s41467-022-29499-8 (PMC8991228; doi:10.1038/s41467-022-29499-8)
Supplement: Supplementary file 1 — Supplementary Information [file 41467_2022_29499_MOESM1_ESM.docx]

**Table S1. Composition of Diets**

| **Research Diets Cat No: Ingredient(g)** | **D11092301 5% Casein** | **D11051801 20% Casein** | **D11092308 HF-5%C** | **D11092309 HF-20%C** |
| --- | --- | --- | --- | --- |
| Casein | 50 | 200 | 50 | 200 |
| L-Cystine | 0.75 | 3 | 0.75 | 3 |
| Corn Starch | 485 | 375.7 | 134.1 | 0 |
| Maltodextrin 10 | 150 | 125 | 125 | 125 |
| Sucrose | 107.1 | 107.1 | 107.1 | 107.1 |
| Cellulose | 50 | 50 | 50 | 50 |
| Soybean Oil | 25 | 25 | 25 | 25 |
| Lard | 75 | 75 | 242 | 242 |
| Mineral Mix S10022C | 3.5 | 3.5 | 3.5 | 3.5 |
| Calcium Carbonate | 8.7 | 12.5 | 8.7 | 12.495 |
| Calcium Phosphate Dibasic | 5.3 | 0 | 5.3 | 0 |
| Potassium Citrate | 2.4773 | 2.5 | 2.4773 | 2.4773 |
| Potassium Phosphate | 6.86 | 6.86 | 6.86 | 6.86 |
| Sodium Chloride | 2.59 | 2.59 | 2.59 | 2.59 |
| Vitamin Mix V10037 | 10 | 10 | 10 | 10 |
| Choline Bitrartrate | 2.5 | 2.5 | 2.5 | 2.5 |
| FD&C Yellow Die #5 | 0 | 0.05 | 0 | 0.025 |
| FD&C Red Dye #40 | 0.05 | 0 | 0.025 | 0 |
| FD&C Blue Dye #1 | 0 | 0 | 0.025 | 0.025 |
| **Total** | 984.8 | 1001.3 | 775.9 | 792.6 |
|  |  |  |  |  |
|  | **D11092301** | **D11051801** | **D11092308** | **D11092309** |
| **Ingredient(g)** | **5% Casein** | **20% Casein** | **HF-5%C** | **HF-20%C** |
| **gm%** |  |  |  |  |
| Protein | 5 | 18 | 6 | 23 |
| Carbohydrate | 76 | 62 | 48 | 31 |
| Fat | 10 | 10 | 34 | 34 |
| **kcal%** |  |  |  |  |
| Protein | 4 | 18 | 4 | 18 |
| Carbohydrate | 74 | 60 | 37 | 24 |
| Fat | 22 | 22 | 59 | 59 |

**Table S2. List of Primers**

| **Oligonucleotides** | |
| --- | --- |
| *Fgf21* Forward: CAAATCCTGGGTGTCAAAGC | Integrated DNA Technologies (IDT) |
| *Fgf21* Reverse: CATGGGCTTCAGACTGGTAC | IDT |
| *Fgfr1* Forward: AAAGATCTGGTATCCTGTGCC | IDT |
| *Fgfr1* Reverse: TCGAGCTAAGCCAAAGTCTG | IDT |
| *Klb* Forward: CAGGGATATCTACATCACAGCC | IDT |
| *Klb* Reverse: GTAGCCTTTGATTTTGACCTTGTC | IDT |
| *Fas* Forward: GGGATCTGGTGAAAGCTGTAG | IDT |
| *Fas* Reverse: GTGTTCTCGTTCCAGGATCTG | IDT |
| *Scd-1* Forward: CTGTACGGGATCATACTGGTTC; | IDT |
| *Scd-1* Reverse: CGTGCCTTGTAAGTTCTGTG | IDT |
| *Srebp1* Forward*:* AGATTGTGGAGCTCAAAGACC | IDT |
| *Srebp1* Reverse: CACTTCGTAGGGTCAGGTTC | IDT |
| *Ucp1* Forward: CACCTTCCCGCTGGACAC | IDT |
| *Ucp1* Reverse: CCCTAGGACACCTTTATACCTAATGG | IDT |
| *Phgdh* Forward: ATTGTCGGCCTTCTGAGAGA | IDT |
| *Phgdh* Reverse : AAGACAGCTCCGTTGAGCAT | IDT |
| *ASNS* Forward: GGAGAGGGGTCAGATGAACTT | IDT |
| *ASNS* Reverse: CTCCTCCTCGGCCTTCTC | IDT |
| *AdipoQ* Forward: TGTCTGTACGATTGTCAGTGG | IDT |
| *AdipoQ* Reverse: AGTAACGTCATCTTCGGCATG | IDT |
| ppar-a Forward: CTGAACATCGACTGTCGAA | IDT |
| ppar-a Reverse: GCTCTCCATGTCATGTATGA | IDT |
| ppar-g Forward: TGTTATGGGTGAAACTCTGGG | IDT |
| ppar-g Reverse: CCAACTTCGGAATCAGCTCT | IDT |
| pgc-1 Forward: CCCTGCCATTGTTAAGACC | IDT |
| pgc-1 Reverse: TGCTGCTGTTCCTGTTTTC | IDT |
| *Cidea* Forward: ATCACAACTGGCCTGGTTACG | IDT |
| *Cidea* Reverse: TACTACCCGGTGTCCATTTCT | IDT |


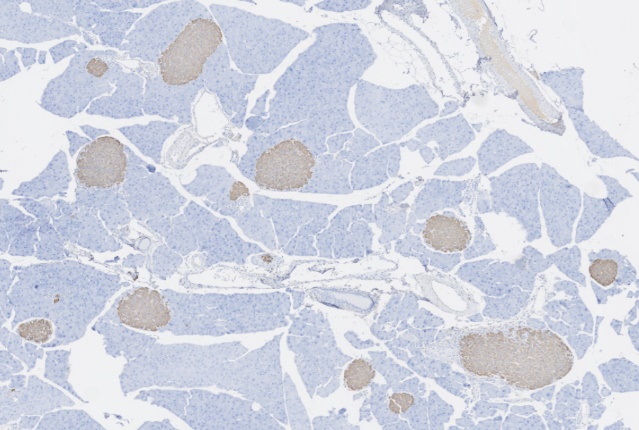


WT-CON

WT-LP


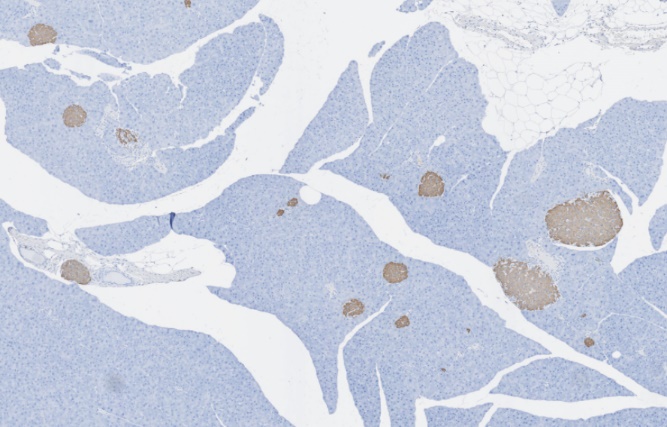

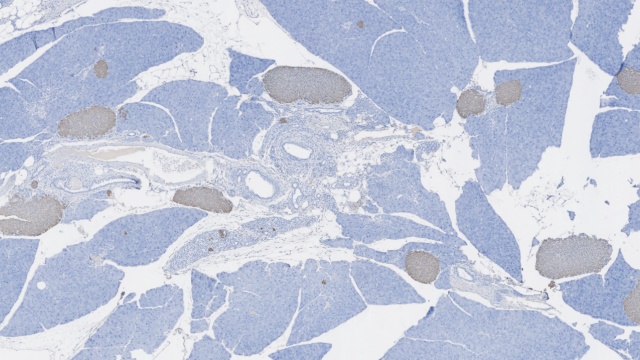


*Fgf21* KO-CON


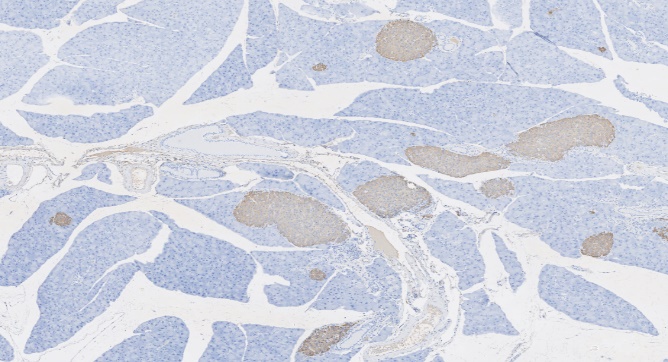


*Fgf21* KO-LP

**Supplemental Figure S1**

Representative images of pancreatic tissue highlighting islet numbers and size, associated with insulin positive area and islet fraction data provided in Figures 4K and 4L. Final data in Figure 4 reflect the averaging of at least 10 sections per animal, with 5 animals per treatment group being used for final statistical analysis. Scale bar represents 500um.

WT-CON

*Fgf21* KO-CON

**
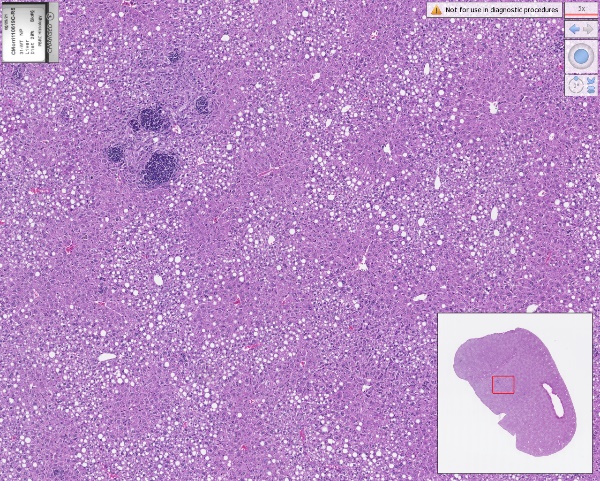

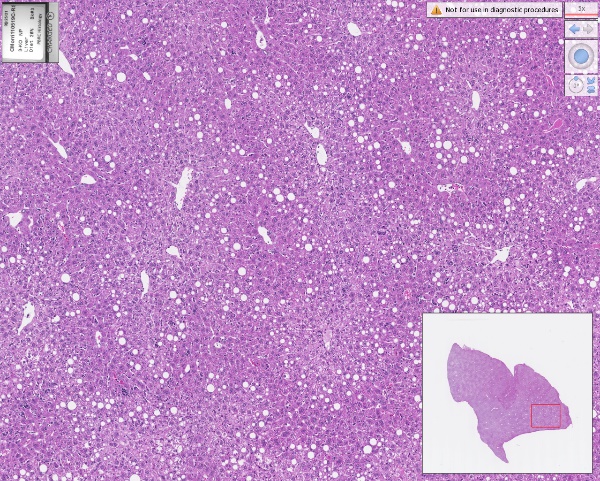
**

*Fgf21* KO-LP

WT-LP

**
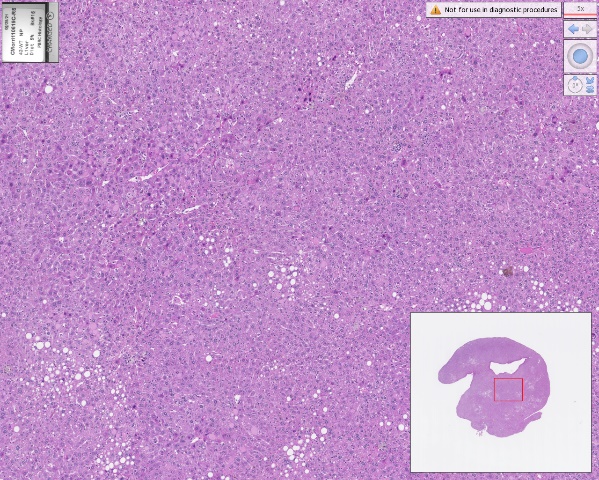
**
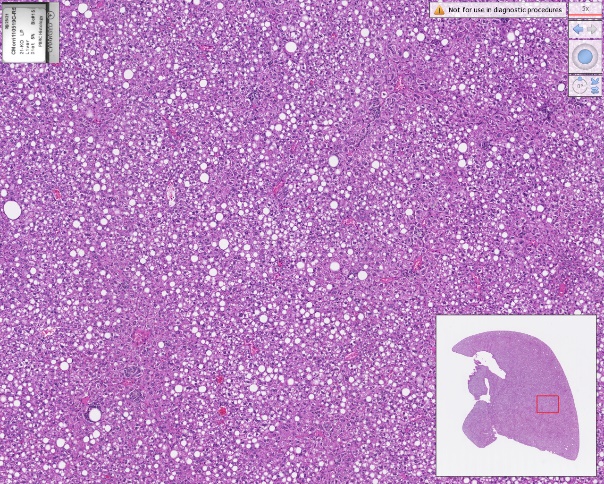


**Supplemental Figure S2**

Representative images of liver H&E staining in mice from the metabolic cohort, collected at sacrifice at 22 months of age. Images are for illustrative purposes and were not used in any subsequent analysis. Scale bar represents 500um.

*Fgf21* KO-CON
