# Supplementary material for: FGF21 is required for protein restriction to extend lifespan and improve metabolic health in male mice
Source: Nat Commun. 2022 Apr 7;13:1897. doi: 10.1038/s41467-022-29499-8 (PMC8991228; doi:10.1038/s41467-022-29499-8)
Supplement: Supplementary file 2 — Reporting Summary [file 41467_2022_29499_MOESM2_ESM.pdf]

nature portfolio

Corresponding author(s): Cristal Hill; Christopher Morrison

Last updated by author(s): Dec 17, 2012

Reporting Summary

Nature Portfolio wishes to improve the reproducibility of the work that we publish. This form provides structure for consistency and transparency in reporting. For further information on Nature Portfolio policies, see our Editorial Policies and the Editorial Policy Checklist.

Statistics

For all statistical analyses, confirm that the following items are present in the figure legend, table legend, main text, or Methods section.

n/a	Confirmed	
☐	☒	The exact sample size (n) for each experimental group/condition, given as a discrete number and unit of measurement
☐	☒	A statement on whether measurements were taken from distinct samples or whether the same sample was measured repeatedly
☐	☒	The statistical test(s) used AND whether they are one- or two-sided<br>*Only common tests should be described solely by name; describe more complex techniques in the Methods section.*
☐	☒	A description of all covariates tested
☐	☒	A description of any assumptions or corrections, such as tests of normality and adjustment for multiple comparisons
☐	☐	A full description of the statistical parameters including central tendency (e.g. means) or other basic estimates (e.g. regression coefficient) AND variation (e.g. standard deviation) or associated estimates of uncertainty (e.g. confidence intervals)
☐	☒	For null hypothesis testing, the test statistic (e.g. F, t, r) with confidence intervals, effect sizes, degrees of freedom and P value noted<br>*Give P values as exact values whenever suitable.*
☒	☐	For Bayesian analysis, information on the choice of priors and Markov chain Monte Carlo settings
☒	☐	For hierarchical and complex designs, identification of the appropriate level for tests and full reporting of outcomes
☒	☐	Estimates of effect sizes (e.g. Cohen's d, Pearson's r), indicating how they were calculated

Our web collection on statistics for biologists contains articles on many of the points above.

Software and code

Policy information about availability of computer code

Data collection	No software was used for data collection
Data analysis	Data were analyzed using the SAS version 9 or JMP Pro version 16 software packages (SAS Institute)

For manuscripts utilizing custom algorithms or software that are central to the research but not yet described in published literature, software must be made available to editors and reviewers. We strongly encourage code deposition in a community repository (e.g. GitHub). See the Nature Portfolio guidelines for submitting code & software for further information.

Data

Policy information about availability of data

All manuscripts must include a data availability statement. This statement should provide the following information, where applicable:

- Accession codes, unique identifiers, or web links for publicly available datasets
- A description of any restrictions on data availability
- For clinical datasets or third party data, please ensure that the statement adheres to our policy

All data generated in this study are provided in the Source Data file, and from the corresponding author upon reasonable request

Field-specific reporting

Life sciences study design

All studies must disclose on these points even when the disclosure is negative.

Sample size	Sample sizes were generated based on power calculations as well as prior experience with aging and metabolic studies
Data exclusions	No data was excluded from the analysis
Replication	The nature of lifespan or aging studies makes replication of the in vivo work impractical, with the lifespan study requiring over 3 years and the metabolic study 2 years to complete the animal component. For various endpoints, we have attempted to replicate key findings by measuring multiple genes or endpoints within a specific biological niche, for instance lipid metabolism or thermogenesis, to provide some measure of replication.
Randomization	All animal were randomized to their respective treatment groups.
Blinding	Blinding was not possible during in vivo data collection, as diet composition was apparent within the cage and mouse genotype is required on the cage card. Individuals conducting subsequent tissue and molecular biology analysis (RNA extraction, qPCR, etc) were blinded to groups, as they only were aware of individual animal IDs.

Reporting for specific materials, systems and methods

We require information from authors about some types of materials, experimental systems and methods used in many studies. Here, indicate whether each material, system or method listed is relevant to your study. If you are not sure if a list item applies to your research, read the appropriate section before selecting a response.

Materials & experimental systems

n/a	Involved in the study	
☐	☒	Antibodies
☒	☐	Eukaryotic cell lines
☒	☐	Palaeontology and archaeology
☐	☒	Animals and other organisms
☒	☐	Human research participants
☒	☐	Clinical data
☒	☐	Dual use research of concern

Methods

n/a	Involved in the study	
☒	☐	ChIP-seq
☒	☐	Flow cytometry
☒	☐	MRI-based neuroimaging

Antibodies

Antibodies used	Guinea pig anti-insulin (1:800, #18-0067, Invitrogen, Grand Island, NY) with secondary detection performed using HRP-conjugated rabbit anti-guinea pig (1:800, A5545, Sigma, Saint Louis, MO) was used to label pancreatic islets
Validation	The Invitrogen guinea pig anti-insulin antibody is well established to bind insulin. It has been used in multiple studies to bind insulin and colocalize with the pancreatic beta-cells, and has been confirmed via genetic knockout/downregulation experiments. PMIDs: 23217255 and 29914854

Animals and other organisms

Policy information about studies involving animals; ARRIVE guidelines recommended for reporting animal research

Laboratory animals	Male mice on the C57BL6 background were used in this study. Mice were started on diets at 3 months of age until natural death, or at 12 months of age until 16 months. Animals were used in 12:12 light/dark room in standard shoebox cages with standard room-level temperature and humidity controls
Wild animals	No wild animals were used
Field-collected samples	No field collected samples
Ethics oversight	All work was approved by the Pennington Biomedical Research Center Institutional Animal Care and Use Committee

Note that full information on the approval of the study protocol must also be provided in the manuscript.
